# A Synoptic Account of Flora of Solapur District, Maharashtra (India)

**DOI:** 10.3897/BDJ.3.e4282

**Published:** 2015-01-16

**Authors:** Krushnadeoray U. Garad, Ramchandra D. Gore, Sayajirao P. Gaikwad

**Affiliations:** † Life Science Research Laboratory, Walchand College of Arts and Science, Solapur- 413 006 (MS), Solapur, India

**Keywords:** Flora, Taxonomy, Solapur, Maharashtra

## Abstract

The present paper provides the first systematic and comprehensive account of the flora of Solapur district of Maharashtra (India). The flora of this region demonstrates a wide range of species diversity and growth forms. The vegetation of the district mainly represents tropical dry deciduous forests, thorny open scrub and vast grasslands. During the present work, a total of 1441 taxa belonging to 699 genera and 125 families of flowering plants were recorded. A new species *Crinum solapurense* Gaikwad *et al.* is described. Fabaceae is the dominant family with 210 taxa, followed by Poaceae (157 taxa), Asteraceae (85 taxa), Malvaceae (68 taxa) and Euphorbiaceae (48 taxa). *Acacia* is the largest genus with 25 taxa, followed by *Euphorbia* (23), *Cyperus* (22), *Crotalaria* (19) and *Ipomoea* (19). The herbaceous flora of the district is notable as it amounts to 56.21% of the whole of flora. The ratio of indigenous woody to herbaceous components is 1:1.28. The proportion of indigenous taxa (978) to the cultivated ones (460) is 1.35: 0.5 in the district.

## Introduction

The Solapur district in India is named after its town headquarter 'Solapur' believed to be derived from two words '*sola*' meaning sixteen and '*pur*' meaning village. The district is predominantly an agrarian tract endowed with a variety of natural resources in the plains of Bhima, Sina and Man Rivers. The climate of the district, in general is dry and extreme. The vegetation is divided into tropical dry deciduous forests (Champion and Seth 1968), the open scrub forests and vast grasslands. The dry deciduous forests and thorny scrub forests play a vital role in the local ecosystem by performing many ecological functions such as ground-water recharge, flood control, retention of nutrients and sediments, and provide habitat for a large number of birds, insects, mammals, reptiles, spiders and microbes. The grasslands of the district are unique and popularly known as Indian Savannas, famous for bird diversity. A hundreds of bird species including a critically endangered Indian Bustard (*Ardeotis nigriceps*) inhabit in these grasslands. Thus, Solapur district is rich in biological diversity but remains rather neglected with regard to its plant wealth.

In Maharashtra, the recorded history suggests that the plant taxonomists were attracted by the forests of Western Ghats. Surprisingly, none had shown any interest in the plants of drought prone region of Maharashtra in general and of Solapur district in particular. There is absolutely no plant collections for the Solapur district anywhere. The botanical exploration in the district remained rather neglected. In spite of high plant diversity and luxuriant vegetation, stray references are found to the plants of the district in well known floras such as 'Bombay Flora' (Dalzell and Gibson 1861) 'Flora of the Presidency of Bombay' (Cooke 1908) and 'Forest Flora of Bombay Presidency and Sind' (Talbot 1909, Talbot 1911). Further, these collections do not precisely indicate the localities. In addition in many cases important field notes are lacking. Much of these collections are hardly available in Indian herbaria such as BSI, BLAT, CAL etc. Though State floras like Flora of Maharashtra by Almeida (1996), Almeida (1998), Almeida (2001a), Almeida (2001b), Almeida (2003a), Almeida (2003b), Almeida (2009a) and Almeida (2009b); BSI's Flora of Maharashtra State: monocotyledons by Lakshminarasimhan (1996) and Flora of Maharashtra State: dicotyledons by Singh and Karthikeyan (2000), Singh and Karthikeyan (2001), had been published, they are not directly connected with the flora of Solapur district as they dealt with Maharashtra State of which Solapur district happens to be part. Further, there are a few scattered publications on the plants of Solapur district (e.g. Hemadri 1970; Pathak 2006; Dalave et al. 2010; Suryavanshi and Bachaulkar 2011; Tembhurne and Nanir 2012; Das Das and Singh 2012​; Gaikwad et al. 2012; Gaikwad et al. 2014; Gaikwad et al. 2014a), which are far from satisfactory. Hence, the present work was necessary to get a comprehensive and dependable floristic survey of Solapur district.

### Study area

**Figure 1.**
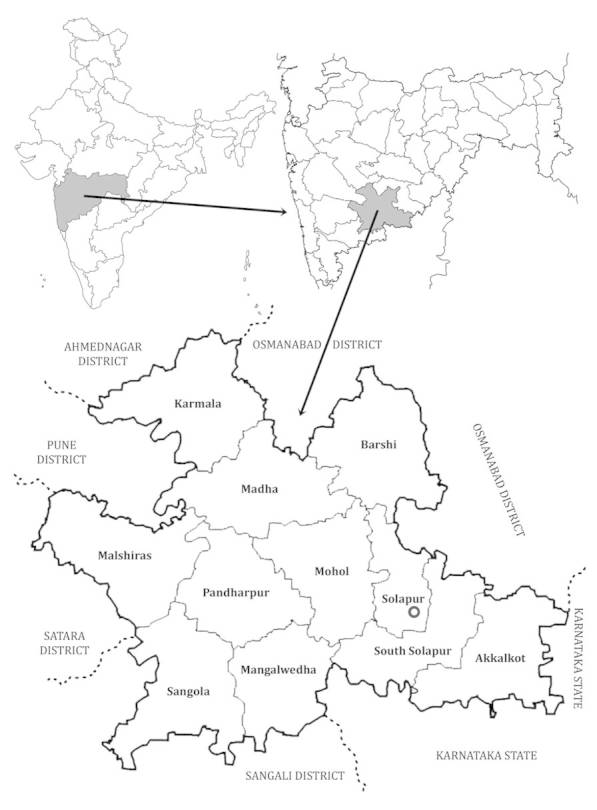
Location map of Solapur district of Maharashtra, India.

The district of Solapur lies between 17°10' N - 18°32' N and 74°42' E - 76°15' E. The district is fairly well defined to its west as well as to its east by the inward-looking scarps of Phaltan Range and the Osmanabad Plateau respectively. Though of an irregular shape, the district is roughly square, 200 km east - west and 150 km north - south. The district has a total area of 14,844.6 sq km and it is divided into eleven-revenue tahsils (Fig. 1). The district is about 550 m above mean sea level. The entire district has the rock type composed of basaltic lava-flows, which erupted in the Cretaceous-Eocene age and a popularly known as Deccan traps (Pathak 2006). The presence of thin mantle of black cotton soil almost everywhere on basalts, river alluvium, sands, gravels, slits and clays represent recent deposits. Calcareous concretions and nodules commonly found in the soil are concentrated near stream courses. The chief rivers of the district are the Bhima, its left-bank feeder the Sina and its right-bank feeders the Nira and the Man. Besides, a good number of lesser streams form the tributaries of the Bhima and serve as its local feeders. The climate of Solapur district is overall agreeable and characterized by general dryness in the major part of the year. The rainfall in the district varies from 448.8 mm (17.67") to 689.2 mm (27.14"). The rainfall during the south-west monsoon in the months of June to September amounts to about 74% of the annual rainfall. September is the rainiest month. The whole district experiences extremes of climate with temperature going down to 5° C in winter and rising up to 45° C in summer. The air is highly humid from June to September (monsoon season) and mostly dries during the rest of the year. The driest part of the year is from March to May when the humidity is between about 20 and 25% on the average in the afternoons. The climate of the region supports the vegetation that can be conveniently divided into tropical dry deciduous forests, thorny forests and vast tracts of grasslands (Champion and Seth 1968; Dikshit 2001​). A population of the district is 43, 17,756 (as per 2011 Census), which constitute 3.84% of the State figures.

## Materials and methods

During the period of five years i.e. from 2009 to 2013, field visits of 2–3 days duration were undertaken to collect plants from selected localities. During the present study, a total of 1441 field numbers comprising about 4467 specimens were collected and deposited in the Herbarium of Walchand College of Arts and Science, Solapur (MS), India. While carrying out the floristic survey, every effort was made to collect the plants in all three seasons viz. pre-monsoon (April–May), monsoon (June–September) and post-monsoon (October–March). Special attention was paid to under or unexplored remote areas of the district. In addition to the collection of wild plants, the efforts were made to collect weeds, which cover a wide range of ecological habitats. Cultivated plants have also been collected as the work was undertaken to study the flora of the district. All the collected specimens processed for drying by using regular drying method with blotting papers and newspapers (Santapau 1955, Jain and Rao 1960, Rao and Sharma 1990). Most of the specimens and field identifications confirmed satisfactorily with the help of available literature (Chakravarty 1982, Sanjappa 1991, Sharma and Balakrishnan 1993, Sharma and Sanjappa 1993, Sharma et al. 1993, Hajra et al. 1995a, Hajra et al. 1995b, Cook 1996, Almeida 1996, Almeida 1998, Almeida 2001a, Almeida 2001b, Almeida 2003a, Almeida 2003b, Almeida 2009a, Almeida 2009b, Naik 1998, Jagtap and Singh 1999, Singh et al. 2000, Singh and Karthikeyan 2000, Singh and Karthikeyan 2001, Mishra and Singh 2001, Singh 2001a, Singh 2001b, Dutta and Deb 2004, Balakrishnan and Chakrabarty 2007, Ansari 2008, Ansari and Balakrishnan 2009, Binojkumar and Balakrishnan 2010, Yadav 2010, Potdar et al. 2012). Doubtful identifications of the specimens were confirmed by comparing them with authentically identified specimens deposited in the Herbarium of Botanical Survey of India, Pune (BSI); Blatter Herbarium, St. Xavier’s college, Mumbai (BLAT) and Dr. Babasaheb Ambedkar Marathwada University Herbarium, Aurangabad (BAMU). Some important plants are featured in photographs (Figs 2, 3, 4).

## Analysis

**Results**

In the present work, authors have provided information on the floristic diversity of Solapur district of Maharashtra, India for the first time. A total of 1441 taxa (including infraspecific ranks) belonging to 699 genera and 125 families of flowering plants have been recorded of which about 860 species are documented for the first time. An assessment of the total constituents of the flowering plants of the district shows that the Core eudicots taxa outnumber those of the monocots. The herbaceous taxa of the district is notable as it amounts to 56.21% of the whole of flora. Fabaceae and Poaceae are dominant in herbaceous vegetation in terms of number of species and frequency percentages indicating favorable climatic and edaphic factors for agriculture in the district. *Aristida*, *Cenchrus*, *Chloris*, *Cymbopogon*, *Cynodon*, *Dichanthium*, *Dactyloctenium*, *Dinebra*, *Eragrostis*, *Euclasta*, *Heteropogon*, *Lophopogon*, *Melanocenchris*, *Mnesithea*, *Paspalum*, *Sehima*, *Setaria* and *Themeda* are dominant grass genera in the study area. *Acacia catechu*, *A. chundra*, *A. leucophloea*, *A. nilotica*, *Bauhinia racemosa*, *Butea monosperma*, *Capparis divaricata*, *C. grandis*, *Euphorbia tirucalli*, *Lannea coromandelica*, *Morinda citrifolia*, *M. coreia* and *Ziziphus caracutta* are common trees in Solapur district whereas *Hardwickia binata*, *Lagerstroemia parviflora*, *Miliusa tomentosa*, *Terminalia arjuna*, *T. bellirica*, *Pterocarpus marsupium* and *Wrightia arborea* are rare in occurrence. In spite of dry or semi-arid general climate of the district, the notable amount of climbers/twiners (10%) occur in Solapur district. Some of them are *Aspidopterys cordata*, *Boerhavia boissieri*, *Clitoria ternatea*, *Cocculus hirsutus*, *Ctenolepis garcinii*, *Diplocyclos palmatus*, *Jasminum auriculatum*, *Mucuna pruriens*, *Operculina turpethum*, *Passiflora edulis*, *Tinospora sinensis* and *Ventilago denticulata*. Solapur district harbors great deals of wealth of vegetables, cereals, pulses and fruit crops due to its varied climatic and edaphic conditions. Mangalveda tahsil of the district is popularly known as store-house of Jawar (*Sorghum*) of the Maharashtra State. About 15 local varieties of *Sorghum* are traditionally cultivated in the district under different local names viz. Boru, Dagadi, Gulbhendi, Kakla, Kuch-kuchi, Lal-jawari, Maldandi, Pawli, Shalu, Tambadi-Jawari, Vandi etc. These varieties are important genome of *Sorghum* crop.

Most of the talukas except some part of Barshi taluka depict thorny scrub forest; the barren hills of this area should be afforested with deciduous elements, which are wild, indigenous and adaptable to dry climate. Solapur district has the forest area about 0.94% (Pathak 2006​) of the total area i.e. about 21.06% less than the minimum requirement (22%), indicating how fast the deterioration of forest has taken place. If the destruction of the vegetation is not checked at this crucial juncture, then it is certainly heading towards natural calamities like drought and flood. The heavy grazing and anthropogenic activities along with uneven pattern of rainfall and frequent droughts for successive years are major causes for deterioration of forest in the district.

In conclusion, therefore, it may be said that the district has a far better potential of plant wealth than was supposed earlier and needs a further careful investigation.

## Discussion

The plants of Solapur district shows wide range of species diversity and growth forms (Tables 1, 2, 7). The herbaceous flora of the district is notable as it amounts to 56.21% of the whole flora. A total of 1441 species including infraspecific taxa distributed over 14,844.6 sq km area reveal a relatively high species density (0.096) when compared with the figure given for adjoining Ahmednagar district (0.061) by (Pradhan and Singh 1999​). Therefore, it may be said that Solapur district rich in its plant wealth.

The total number of species (including subspecies, varieties and forma) reported for Maharashtra as on to-day is 3191 (Table 3). For Solapur district, the number of taxa works out to be 1441. Therefore, percentage of flora of Solapur district out of whole flora of Maharashtra will be 45.15.

To evaluate the dominant ten families a comparison has been made between major floras of the Maharashtra State such as 'The Flora of the Presidency of Bombay' (Cooke 1908), 'Flora of Maharashtra: monocotyledons' (Lakshminarasimhan 1996), 'Flora of Marathwada' (Naik 1998), 'Flora of Maharashtra: dicotyledons' (Singh and Karthikeyan 2000, Singh and Karthikeyan 2001) and Flora of Solapur district (present work). The Family Fabaceae including three sub-families viz. Papilionoideae, Caesalpinoideae and Mimosoideae taken as a single combined family Leguminosae (Fabaceae), as done by Hooker in his Flora of British India, then that family stands first in the list with 66 genera and 210 species and Solanaceae (12:33) finds tenth place in the list of dominant families (Table 4). This order of dominance, when compared with above-mentioned three floras, it roughly corresponds to the Flora of Marathwada (Table 5). In both floras, same nine families find a place, of-course, with varying positions. Rubiaceae, which occupies the nineth position, fails to find a place amongst the ten dominant families in Flora Solapur district; on the other hand the family Solanaceae and Apocynaceae which occupy tenth and seventh position in Solapur district could not find a place anywhere among the top ten families in Flora of Marathwada. Further, Asteraceae and Euphorbiaceae are occupied at same positions in the both floras. While, Fabaceae and Poaceae are occupy top positions. These facts indicate more or less similar climatic and edaphic situations in Marathwada region and Solapur district.

The family Fabaceae shares maximum number of prominent genera whereas other families except Cyperaceae have only one prominent genus each (Table 6). In contrast to these prominent genera, there are about 416 genera represented by only one species.

**Figure 5.**
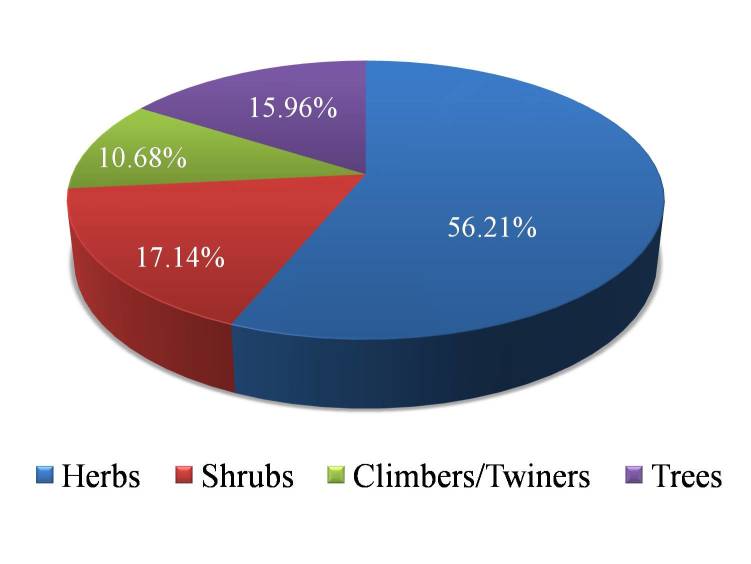
Analysis of life forms of indigenous taxa in flora of Solapur district (Suppl. material 1).

The ratio of indigenous woody (631 taxa) to herbaceous (810 taxa) components is 1:1.28 and that of monocots (244 indigenous taxa) to Magnolids and eudicots (734 indigenous taxa) is 1:3 (Table 7). The floristic spectrum of the district shows very low percentage of phanerophytes and a very high percentage of therophytes. It clearly indicates the drier climatic conditions together with other biological influence (Fig. 5).

Mere 23 endemic plant species occur in Solapur district which include *Hardwickia binata* Roxb. a monotypic tree genera, *Ornithogalum saxorum* (Blatt.) J.C. Manning & Goldblatt a critically endangered species and *Dregea lanceolata* (Cooke) Santapau & Wagh, (Ahmedullah and Nayar 1986, Nayar and Sastry 1987, Nayar 1996, Mishra and Singh 2001, Irwin and Narasimhan 2011, Gaikwad et al. 2014b​).

**Figure 6.**
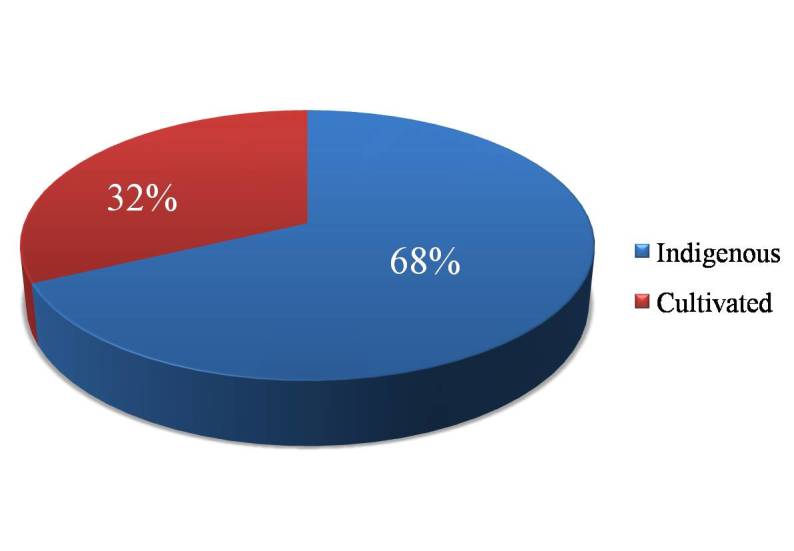
Proportion of indigenous to cultivated taxa (Suppl. material 2).

In the present floristic work, the commonly cultivated species are also included in addition to indigenous ones. The proportion of indigenous taxa (978) to the cultivated ones (460) is 1.35:0.5 (Fig. 6). This high proportion of cultivated species cannot be neglected as it forms the major component of urban flora in particular.

## Supplementary Material

Supplementary material 1 Analysis of life forms of indigenous taxa in flora of Solapur district Data type: Graph
Brief description: Analysis of life forms of indigenous taxa in flora of Solapur district.
File: oo_36180.docx R.D. Gore

Supplementary material 2 Proportion of indigenous to cultivated taxa Data type: Graph
Brief description: Proportion of indigenous to cultivated taxa.
File: oo_36181.docx R.D. Gore

## Figures and Tables

**Figure 2a.**
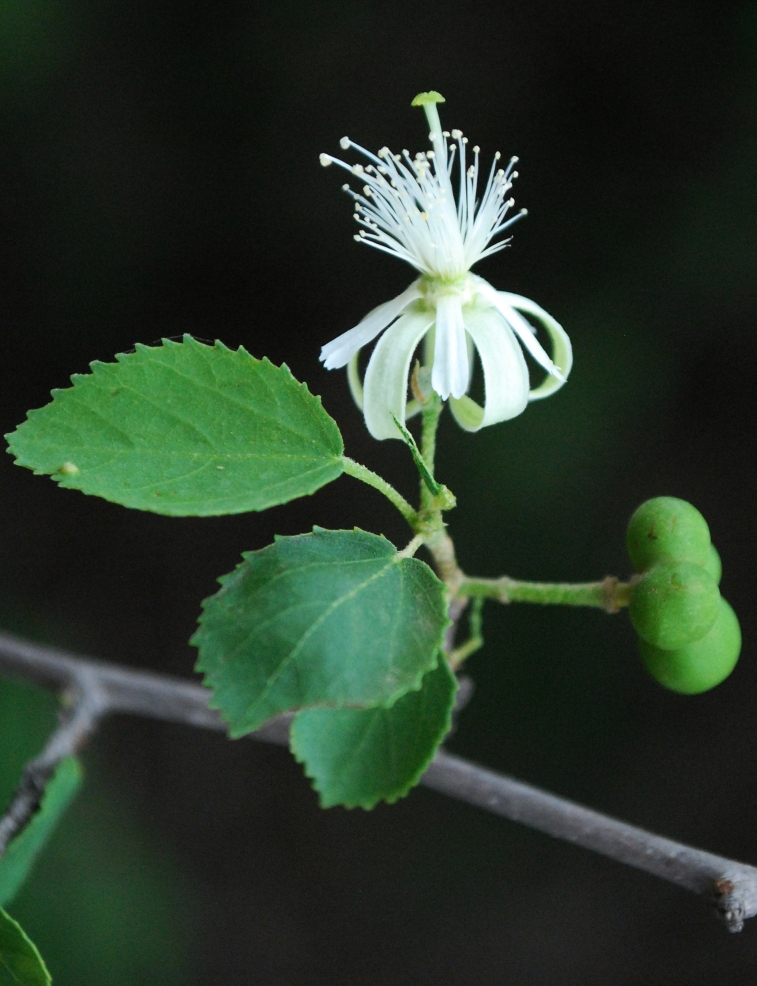
*Grewia tenax* (Forssk.) Fiori

**Figure 2b.**
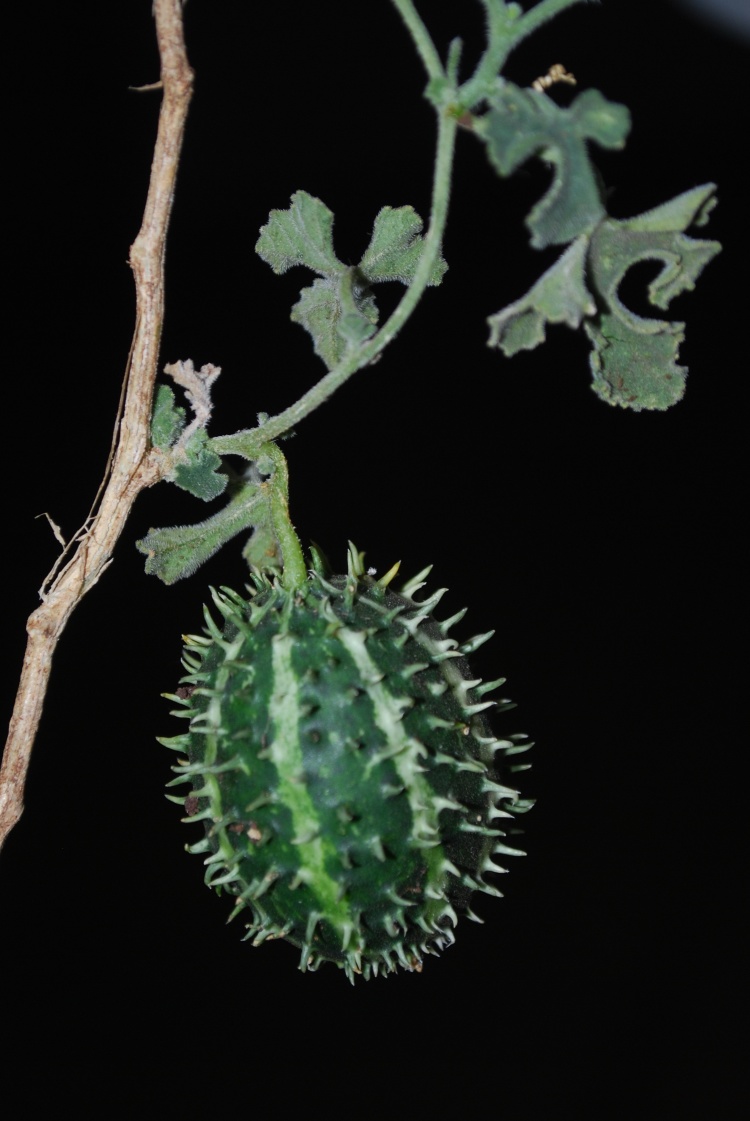
*Cucumis prophetarum* L.

**Figure 2c.**
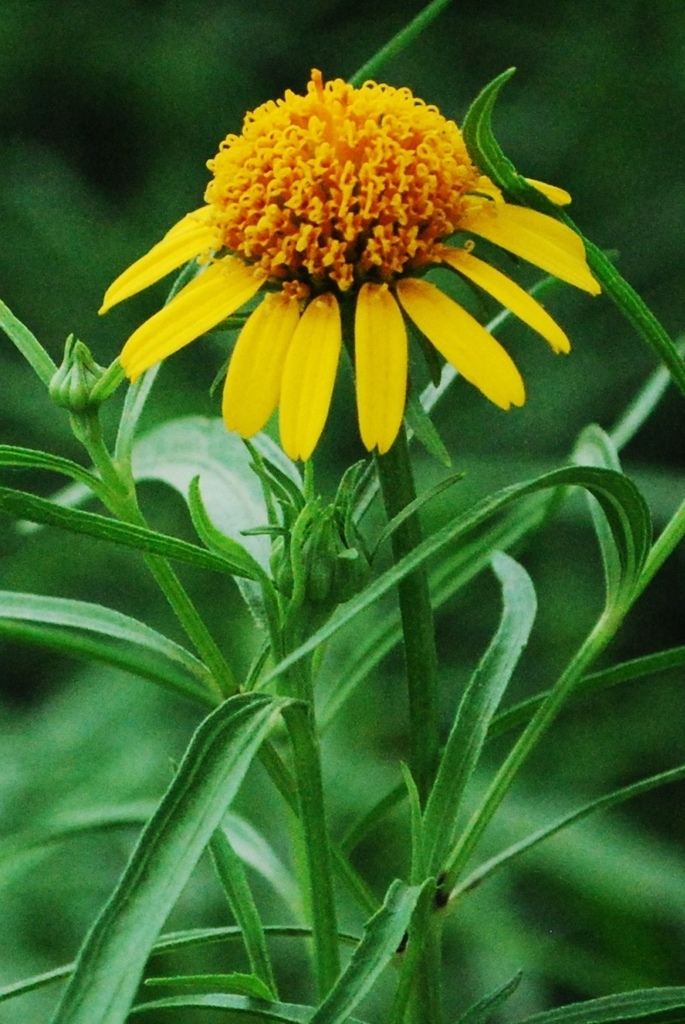
*Pascalia glauca* Ortega

**Figure 2d.**
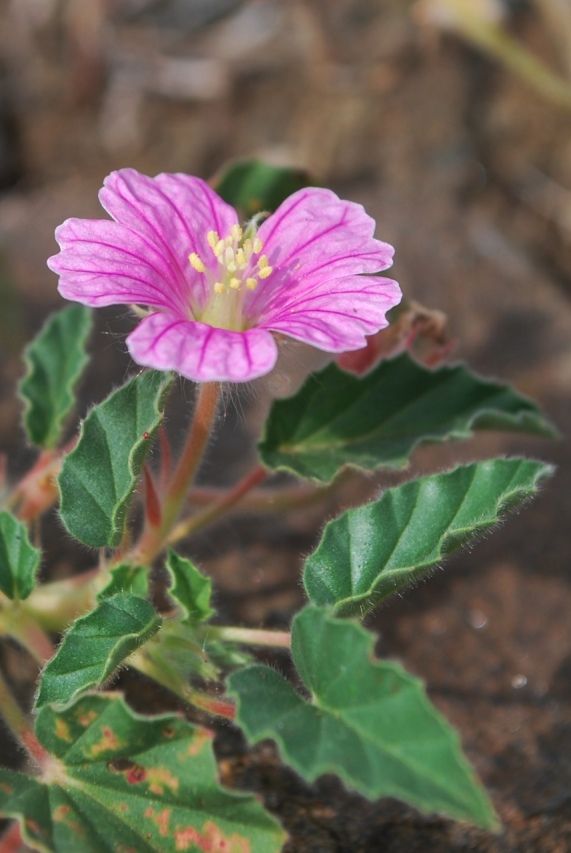
*Monsonia senegalensis* Guill. & Perr.

**Figure 3a.**
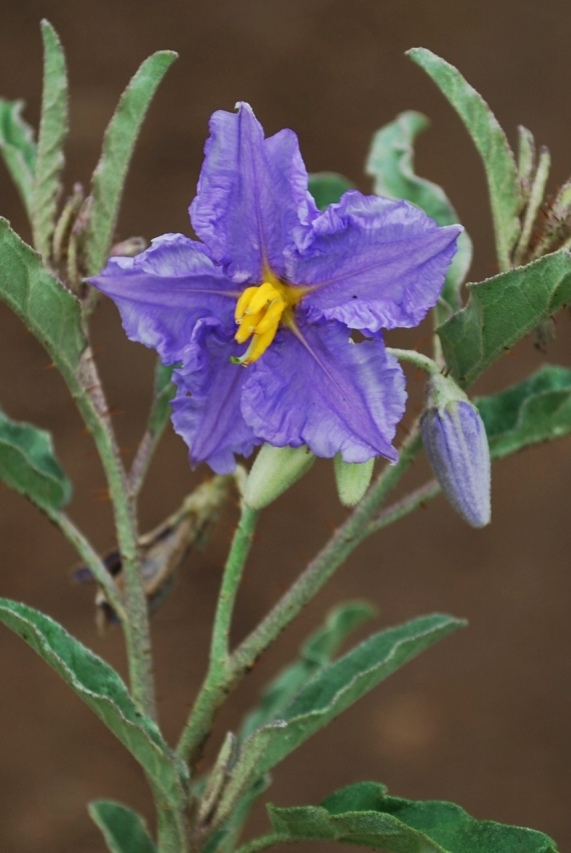
*Solanum elaeagnifolium* Cav.

**Figure 3b.**
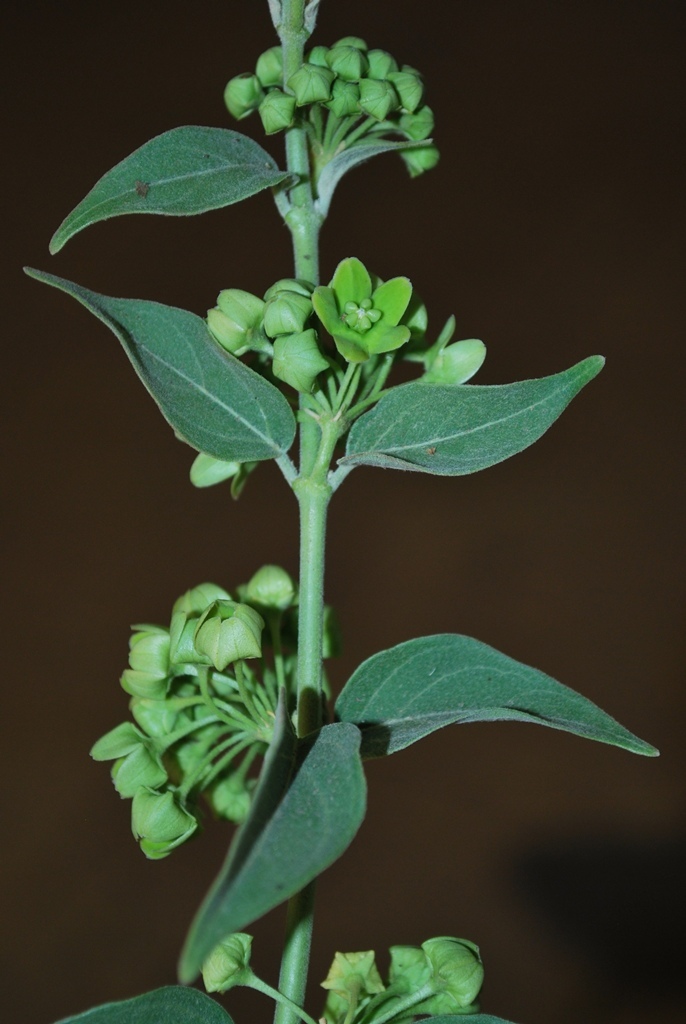
*Dregea lanceolata* (Cooke) Santapau & Wagh

**Figure 3c.**
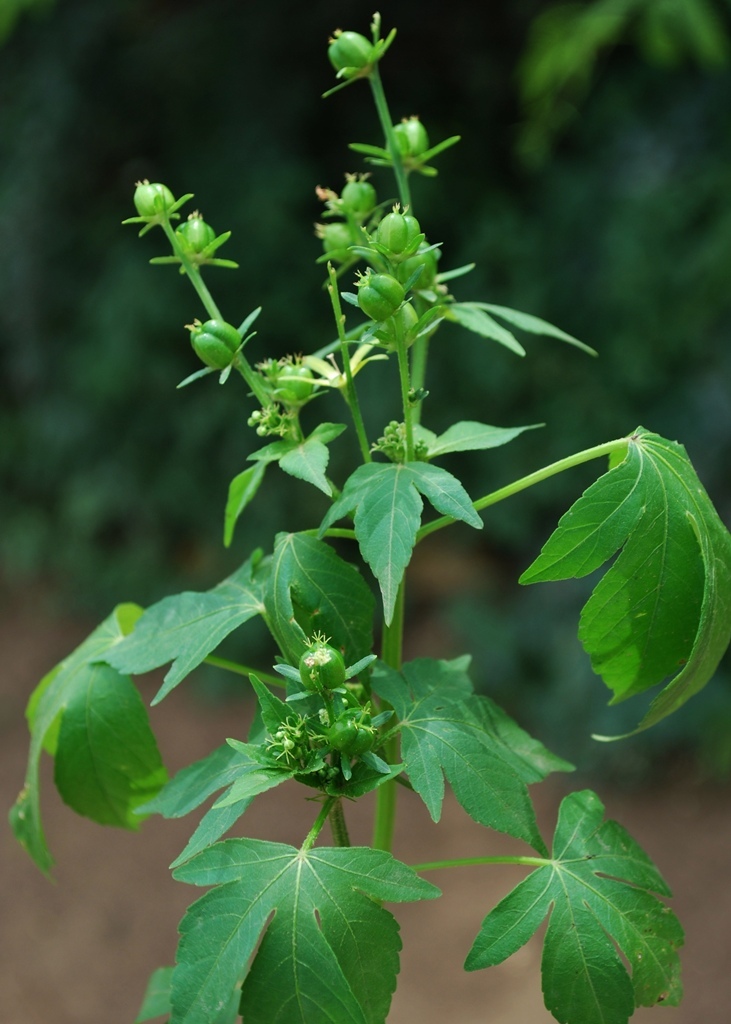
*Astraea lobata* (L.) Klotz.

**Figure 3d.**
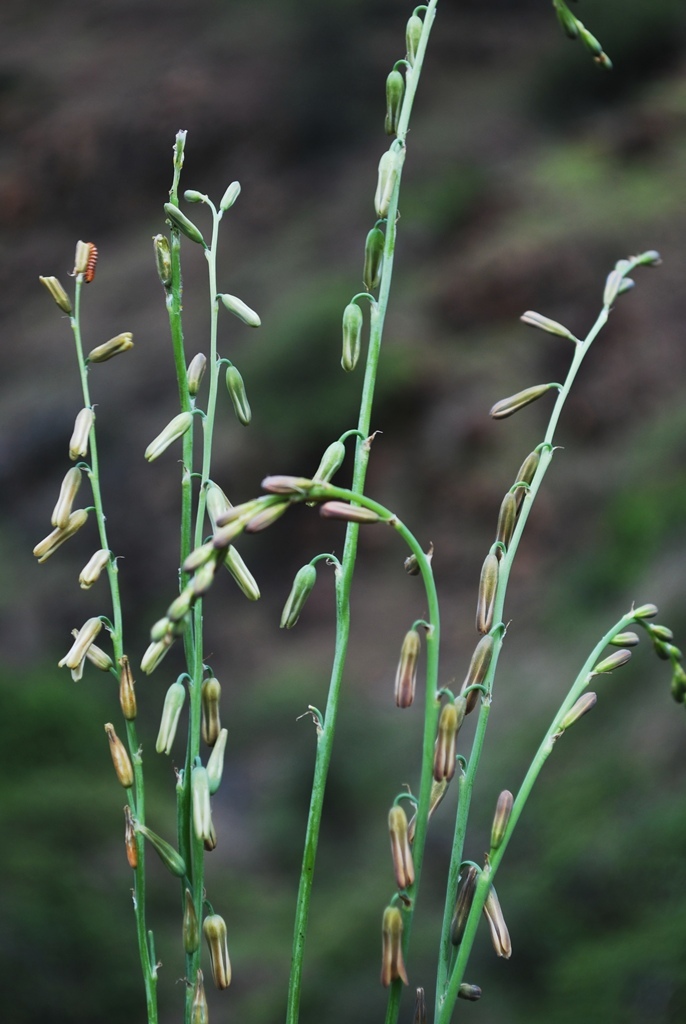
*Dipcadi saxorum* Blatt.

**Figure 4a.**
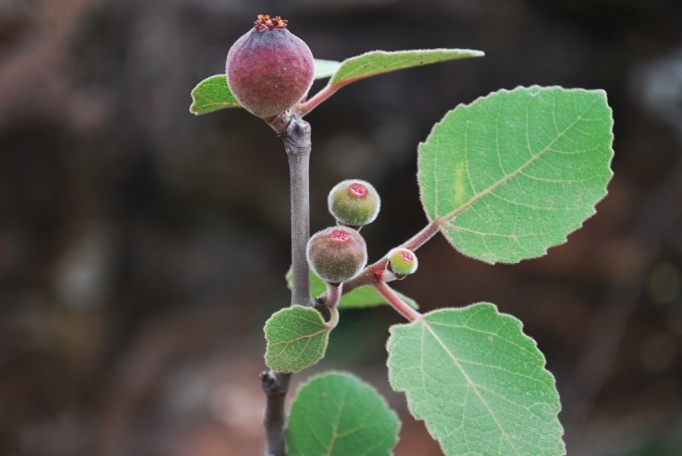
Ficus palmata Forssk subsp. virgata (Roxb.) Browicz

**Figure 4b.**
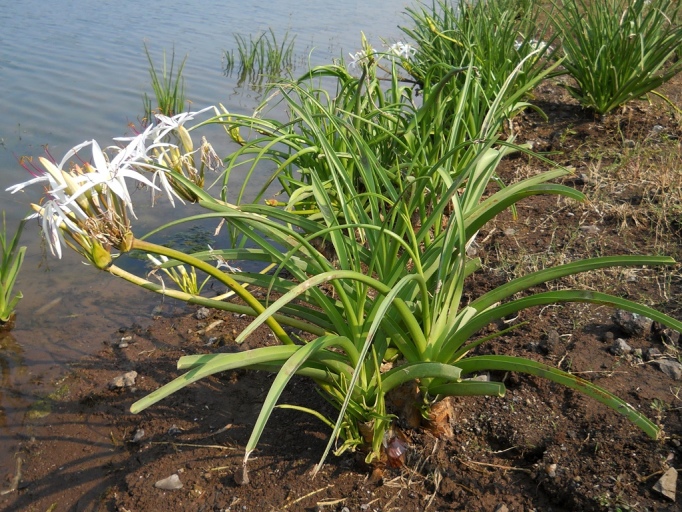
*Crinum solapurense* Gaikwad *et al.*

**Table 1.** List of families with number of genera and species including infraspecific taxa.

Sr. No.	Family	Genera	Species	Subspecies	Variety	Forma	Species including infraspecific taxa
**ANITA Grade**							
1	Nymphaeaceae	1	1	-----	-----	-----	1
**MAGNOLIDS**							
2	Piperaceae	1	2	-----	-----	-----	2
3	Aristolochiaceae	1	1	-----	-----	-----	1
4	Magnoliaceae	1	1	-----	-----	-----	1
5	Annonaceae	4	6	-----	-----	-----	6
**MONOCOTS**							
6	Araceae	14	21	-----	-----	-----	21
7	Hydrocharitaceae	4	6	-----	-----	-----	6
8	Potamogetonaceae	2	3	-----	-----	-----	3
9	Dioscoreaceae	1	1	-----	-----	-----	1
10	Pandanaceae	1	1	-----	-----	-----	1
11	Colchicaceae	2	4	-----	-----	-----	4
12	Smilacaceae	1	1	-----	-----	-----	1
13	Orchidaceae	2	1	-----	1	-----	2
14	Hypoxidaceae	1	1	-----	-----	-----	1
15	Iridaceae	1	1	-----	-----	-----	1
16	Xanthorrhoeaceae	2	2	-----	-----	-----	2
17	Amaryllidaceae	7	13	-----	-----	-----	13
18	Asparagaceae	13	27	-----	3	-----	30
19	Arecaceae	17	22	-----	-----	-----	22
20	Commelinaceae	6	17	-----	-----	-----	17
21	Pontederiaceae	2	3	-----	-----	-----	3
22	Strelitziaceae	1	1	-----	-----	-----	1
23	Heliconiaceae	1	1	-----	-----	-----	1
24	Musaceae	1	1	-----	-----	-----	1
25	Cannaceae	1	3	-----	-----	-----	3
26	Marantaceae	2	2	-----	-----	-----	2
27	Costaceae	1	1	-----	-----	-----	1
28	Zingiberaceae	3	3	-----	-----	-----	3
29	Typhaceae	1	1	-----	-----	-----	1
30	Eriocaulaceae	1	1	-----	-----	-----	1
31	Cyperaceae	11	42	4	1		47
32	Poaceae	68	141	-----	16	-----	157
**EUDICOTS**							
33	Ceratophyllaceae	1	1	-----	-----	-----	1
34	Papaveraceae	3	4	-----	-----	-----	4
35	Menispermaceae	3	3	-----	1	-----	4
36	Ranunculaceae	1	1	-----	-----	-----	1
37	Nelumbonaceae	1	1	-----	-----	-----	1
38	Proteaceae	1	1	-----	-----	-----	1
**CORE EUDICOTS**							
39	Crassulaceae	2	5	-----	-----	-----	5
40	Vitaceae	4	7	-----	-----	-----	7
**CORE EUDICOTS: ROSIDS**							
41	Zygophyllaceae	3	4	-----	-----	-----	4
42	Fabaceae	66	176	8	24	2	210
43	Polygalaceae	1	4	-----	-----	-----	4
44	Rosaceae	1	5	-----	-----	-----	5
45	Rhamnaceae	2	7	-----	-----	-----	7
46	Ulmaceae	1	1	-----	-----	-----	1
47	Cannabaceae	1	1	-----	-----	-----	1
48	Moraceae	3	14	1	-----	-----	15
49	Urticaceae	1	1	-----	-----	-----	1
50	Casuarinaceae	1	1	-----	-----	-----	1
51	Cucurbitaceae	15	21	-----	9	-----	30
52	Begoniaceae	1	1	-----	-----	-----	1
53	Celastraceae	3	3	-----	-----	-----	3
54	Oxalidaceae	3	5	-----	1	-----	6
55	Euphorbiaceae	14	48	-----	-----	-----	48
56	Ochnaceae	1	-----	-----	1	-----	1
57	Phyllanthaceae	4	12	-----	-----	-----	12
58	Elatinaceae	1	1	-----	-----	-----	1
59	Malpighiaceae	4	5	-----	-----	-----	5
60	Putranjivaceae	1	1	-----	-----	-----	1
61	Passifloraceae	2	4	-----	-----	-----	4
62	Salicaceae	3	4	-----	-----	-----	4
63	Violaceae	2	2	-----	-----	-----	2
64	Linaceae	1	2	-----	-----	-----	2
65	Geraniaceae	2	2	-----	-----	-----	2
66	Combretaceae	3	9	-----	-----	-----	9
67	Lythraceae	6	13	-----	-----	-----	13
68	Onagraceae	1	2	-----	-----	-----	2
69	Myrtaceae	4	7	-----	1	-----	8
70	Burseraceae	2	2	-----	-----	-----	2
71	Anacardiaceae	6	6	-----	-----	-----	6
72	Sapindaceae	4	5	-----	-----	-----	5
73	Rutaceae	6	13	-----	-----	-----	13
74	Simaroubaceae	1	1	-----	-----	-----	1
75	Meliaceae	5	7	-----	-----	-----	7
76	Muntingiaceae	1	1	-----	-----	-----	1
77	Malvaceae	27	58	2	8		68
78	Bixaceae	2	2	-----	-----	-----	2
79	Tropaeolaceae	1	1	-----	-----	-----	1
80	Moringaceae	1	1	-----	-----	-----	1
81	Caricaceae	1	1	-----	-----	-----	1
82	Salvadoraceae	1	1	-----	-----	-----	1
83	Capparaceae	3	10	1	-----	-----	11
84	Cleomaceae	1	5	-----	-----	-----	5
85	Brassicaceae	9	10	1	3	-----	14
**CORE EUDICOTS: ASTERIDS**							
86	Santalaceae	2	2	-----	-----	-----	2
87	Tamaricaceae	1	1	-----	-----	-----	1
88	Plumbaginaceae	1	3	-----	-----	-----	3
89	Polygonaceae	5	6	-----	1	-----	7
90	Caryophyllaceae	4	5	-----	-----	-----	5
91	Amaranthaceae	12	27	-----	3	-----	30
92	Aizoaceae	3	4	-----	-----	-----	4
93	Nyctaginaceae	4	6	-----	2	-----	8
94	Molluginaceae	3	7	-----	-----	-----	7
95	Basellaceae	1	1	-----	-----	-----	1
96	Portulacaceae	1	3	2	-----	-----	5
97	Cactaceae	4	4	-----	-----	-----	4
98	Cornaceae	1	-----	1	-----	-----	1
99	Balsaminaceae	1	-----	-----	1	-----	1
100	Polemoniaceae	1	1	-----	-----	-----	1
101	Lecythidaceae	1	1	-----	-----	-----	1
102	Sapotaceae	3	3	-----	2	-----	5
103	Ebenaceae	1	5	-----	-----	-----	5
104	Primulaceae	1	2	-----	-----	-----	2
105	Rubiaceae	17	29	-----	1	-----	30
106	Gentianaceae	5	7	-----	3	-----	10
107	Loganiaceae	1	1	-----	-----	-----	1
108	Apocynaceae	35	42	1	2	-----	45
109	Boraginaceae	4	14	1	2	-----	17
110	Convolvulaceae	11	36	1	-----	3	40
111	Solanaceae	12	30	-----	3	-----	33
112	Oleaceae	2	8	-----	-----	-----	8
113	Gesneriaceae	1	1	-----	-----	-----	1
114	Scrophulariaceae	15	18	-----	3	-----	21
115	Pedaliaceae	1	2	-----	-----	-----	2
116	Lamiaceae	11	26	-----	5	-----	31
117	Orobanchaceae	1	-----	-----	1	-----	1
118	Lentibulariaceae	1	2	-----	-----	-----	2
119	Acanthaceae	22	39	1	3	-----	43
120	Bignoniaceae	17	19	-----	1	-----	20
121	Verbenaceae	12	16	-----	3	-----	19
122	Martyniaceae	1	1	-----	-----	-----	1
123	Asteraceae	57	81	1	3	-----	85
124	Araliaceae	2	6	-----	-----	-----	6
125	Apiaceae	8	9	-----	-----	-----	9

**Table 2.** Showing statistical account of the flora of Solapur district.

Class	Families	Genera	Species	Infraspecific taxa
**ANITA Grade**	01	01	01	---
**MAGNOLIDS**	04	07	10	---
**MONOCOTS**	27	167	321	25
**EUDICOTS**	06	10	11	01
**CORE EUDICOTS**	02	06	12	---
1) Rosids	45	222	480	62
2) Asterids	40	286	486	50
**Total**	**125**	**699**	**1303**	**138**

**Table 3.** Showing comparative account of taxa reported in different taxonomic works on Maharashtra State and Solapur district (*Note*: The figure in the parenthesis indicates account of the Bombay State including Maharashtra, Karnataka and Gujarat, Baluchistan and some part of Rajasthan).

Name of the flora	Families	Genera	Species	Infraspecific taxa
Flora of The Presidency of Bombay (1958 Repr.)	139 (147)	849 (999)	1938 (2513)	94 (162)
Flora of Maharashtra (1996 - 2002)	187	1081	3025	166
The previous literature (1901 - 2012)	-----	-----	578	-----
Flora of Solapur District (Present work)	125	699	1303	138

**Table 4.** Showing ten dominant families in the order of dominance.

**Sr. No.**	**Families**	**Number of taxa**
1.	Fabaceae	210
2.	Poaceae	157
3.	Asteraceae	85
4.	Malvaceae	68
5.	Euphorbiaceae	48
6.	Cyperaceae	47
7.	Apocynaceae	45
8.	Acanthaceae	43
9.	Convolvulaceae	40
10.	Solanaceae	33

**Table 5.** Comparative account of ten dominant families reported in different taxonomic works and Solapur district.

**Flora of the Presidency of Bombay (BSI)** **(1901 - 1908)**	**Flora of Maharashtra State (BSI)** **(1996 - 2001)**	**Flora of Marathwada** **(1998)**	**Flora of Solapur District** **(Present work)**
Fabaceae	Poaceae	Poaceae	Fabaceae
Poaceae	Fabaceae	Fabaceae	Poaceae
Acanthaceae	Cyperaceae	Asteraceae	Asteraceae
Asteraceae	Acanthaceae	Cyperaceae	Malvaceae
Euphorbiaceae	Asteraceae	Euphorbiaceae	Euphorbiaceae
Rubiaceae	Orchidaceae	Acanthaceae	Cyperaceae
Orchidaceae	Euphorbiaceae	Malvaceae	Apocynaceae
Lamiaceae	Rubiaceae	Convolvulaceae	Acanthaceae
Scrophulariaceae	Scrophulariaceae	Rubiaceae	Convolvulaceae
Asclepiadaceae	Malvaceae	Mimosaceae	Solanaceae

**Table 6.** List of top twenty genera represented by higher number of species in the flora of Solapur district.

**Sr. No.**	**Genus**	**Number of taxa**	**Sr. No.**	**Genus**	**Number of taxa**
1.	* Acacia *	25	11.	* Hibiscus *	10
2.	* Euphorbia *	23	12.	* Vigna *	10
3.	* Cyperus *	22	13.	* Leucas *	09
4.	* Crotalaria *	19	14.	* Corchorus *	08
5.	* Ipomoea *	19	15.	* Blumea *	08
6.	* Cassia *	18	16.	* Phyllanthus *	08
7.	* Alysicarpus *	14	17.	* Commelina *	08
8.	* Indigofera *	13	18.	* Desmodium *	07
9.	* Ficus *	13	19.	* Jasminum *	07
10.	* Fimbristylis *	12	20.	* Heliotropium *	07

**Table 7.** Showing analysis of life forms of indigenous taxa.

Life form	Number of species	Percentage (%)
Herbs	810	56.21
Shrubs	247	17.14
Climbers/Twiners	154	10.68
Trees	230	15.96
