# Supplementary material for: A Synoptic Account of Flora of Solapur District, Maharashtra (India)
Source: Biodivers Data J. 2015 Jan 16;(3):e4282. doi: 10.3897/BDJ.3.e4282 (PMC4304262; doi:10.3897/BDJ.3.e4282)
Supplement: Supplementary material 1 — Analysis of life forms of indigenous taxa in flora of Solapur district [file biodiversity_data_journal-3-e4282-s001.docx]

**Fig. 2.** Analysis of life forms of indigenous taxa in flora of Solapur district.
