# Supplementary material for: A Synoptic Account of Flora of Solapur District, Maharashtra (India)
Source: Biodivers Data J. 2015 Jan 16;(3):e4282. doi: 10.3897/BDJ.3.e4282 (PMC4304262; doi:10.3897/BDJ.3.e4282)
Supplement: Supplementary material 2 — Proportion of indigenous to cultivated taxa [file biodiversity_data_journal-3-e4282-s002.docx]

**Fig. 3.** Proportion of indigenous to cultivated taxa.
